# Upper mesophotic reef fish assemblages at Bahía de Banderas, Mexico

**DOI:** 10.3897/BDJ.12.e113125

**Published:** 2024-03-11

**Authors:** Jose Luis Arreola, Cristian Moisés Galván-Villa, Yocanxóchitl Perfecto-Avalos, Fabian Alejandro Rodríguez-Zaragoza, Eduardo Rios Jara

**Affiliations:** 1 Tecnologico de Monterrey, Guadalajara, Mexico Tecnologico de Monterrey Guadalajara Mexico; 2 Departamento de Ecología Aplicada, CUCBA, Universidad de Guadalajara, Zapopan, Jalisco, Mexico Departamento de Ecología Aplicada, CUCBA, Universidad de Guadalajara Zapopan, Jalisco Mexico

**Keywords:** fish refuge, fisheries, icthyofauna, marine fishes, mesophotic zone

## Abstract

There is no information on the species associated with the mesophotic reefs of Banderas Bay, located in the central Mexican Pacific. This study analysed the reef fish assemblage from three depths (50, 60 and 70 m) in three sampling sites of the southern submarine canyon of the Bay: Los Arcos, Bajo de Emirio and Majahuitas. Several analyses were performed to test the hypothesis that there are important differences in fish abundance and species composition between sites and depths. Twenty-two species of bony fishes grouped in 14 families were recorded. PERMANOVA results showed that there were no significant differences in fish diversity parameters between sites, indicating a certain uniformity in their distribution. However, nine species were exclusive to one site and depth (five singleton species with only one individual recorded and four unique species recorded only once). On the other hand, there were significant differences between depths, mainly between 50 and 70 m. Diversity decreases with depth and species composition changes. SIMPER, Shade Plot and NMDS analysis show the most representative species at each depth, with at least half of the species (11) recorded only at 50 m and four species at the deeper levels (60 - 70 m). The observed assemblage includes several of the most caught species in the shallow water artisanal fishery, which is the most traditional and common type of fishery in the Bay. In addition, the *Pomacanthuszonipectus* (Cortés angelfish) is of particular interest, as it has a special protection status in the official Mexican standard (NOM-059-SEMARNAT, 2010) due to its use as an ornamental species in aquaria. We hypothesised that the mesophotic zone may serve as a refuge for these fishes, so we propose that the information obtained is an important basis for new research aimed at the sustainable management of fisheries in the area.

## Introduction

Mesophotic zones are delimited between 30 and 150 m depth and are characterised by low light and low temperature, with a substrate and bottom geomorphology different from those found in shallow zones (Baker et al. 2016, Weiss 2017). Rocky reefs have a global distribution in the mesophotic zones of tropical and subtropical regions. These reefs are now considered to be more extensive than previously thought and it is estimated that they may represent a larger area than shallow reefs (Puglise et al. 2009). They are also considered a valuable resource for the conservation and management of shallow reefs, which are subject to increase due to natural factors (e.g. storms, coral bleaching, changes in acidity and salinity), as well as due to anthropogenic activities and their consequences (e.g. overfishing, tourism, pollution, climate change, introduction of alien species). In the last decade, mesophotic reefs have been proposed as a refuge for shallow reef-dwelling species where they can find a habitat for the development and protection of offspring, food and shelter from surface threats buffered by the effects of depth. Based on this premise, the deep-refuge hypothesis has been proposed, which states that mesophotic reefs can serve as areas that provide conditions to mitigate the negative effects of natural and anthropogenic events in shallow zones, particularly those caused by climate change (Lesser et al. 2009, Bongaerts et al. 2010, Appeldoorn et al. 2016, Semmler et al. 2017, Bongaerts et al. 2019, Bongaerts and Smith 2019, Loya et al. 2019).

Our knowledge of fishes from mesophotic zones comes mostly from studies conducted in the last two decades. These studies have been conducted on coral reefs of Caribbean island systems (Garcia-Sais 2010, Gonzalez-Zapata et al. 2018), the northern Gulf of Mexico (Mayorga-Martínez et al. 2021), Brazilian coasts and islands (Meirelles et al. 2015, Rosa et al. 2016), as well as the Hawaiian Islands (Boland and Parrish 2005, Fukunaga et al. 2016, Pyle et al. 2016, Fukunaga et al. 2017) and islands of the South Pacific and Australia (Abesamis et al. 2018, Coleman et al. 2018, Williams et al. 2019; see Loya et al. (2019)).

The Mexican Pacific coast is influenced by the Eastern Tropical Pacific provinces, in particular the Revillagigedo, Clipperton and Mexican Tropical Pacific eco-regions, as well as the Cortezian eco-region belonging to the NWTP province. (Spalding et al. 2007). To date, two studies have characterised the mesophotic and deep-sea fish assemblages of this province. The first study, by Friedlander et al. (2019), describes marine biodiversity from 0 to 1,500 m depth in Clipperton Atoll, located 1,080 km southwest of Mexico. Using a variety of sampling techniques, they were able to record 96 species from 41 families and 15 orders of fishes, with 67% of the species being distributed at depths < 200 m.

Although Bahía de Banderas is an area of high biodiversity (Moncayo Estrada et al. 2006), there are no published field studies that document the richness of fish species in the mesophotic zone or the bathymetric distribution of the fish assemblage found at depths greater than 30 m. Reef fish are used to exemplify the deep refuge hypothesis and, due to the great information that exists on the fauna of the shallow zones, a comparison can be made with the species found in the mesophotic zone. Generating information and being able to contribute to the deep refuge hypothesis is important to obtain the basic ecological parameters which can then be correlated to the morphology of the environment, the functional variables of the group and the factors of temporality, locality and the different depth levels.

This study aimed to recognise for the first time the diversity of mesophotic reef fishes of Bahía de Banderas in the central Mexican Pacific. In addition, to evaluate the spatial differences in richness and abundance of the mesophotic reef fish group in three localities in the southern part of the Bay. Finally, to describe the bathymetric distribution of fishes at three depth levels (50, 60 and 70 m). This information will help to understand the possible use of the mesophotic zone as a refuge for fishes inhabiting the shallow reefs of the Bay, which is important for the management of coastal fishery resources.

## Material and Methods

### Study area

Bahía de Banderas is located in the central Mexican Pacific, is 40 km long and has an area of more than 1,000 km^2^ (Stokes et al. 2019). The Bay is part of the Mexican Tropical Pacific (PTM) ecoregion and lies on the boundary between the Tropical East Pacific (TEP) and Warm Temperate Northeast Pacific (WTNP) provinces (Spalding et al. 2007). The Bay is considered a transition and convergence zone between the California Current and the North Equatorial Current. Its submarine topography is rugged with significant bathymetric variations. In the north, the average depth is nearly 50 m. In the south, there is a submarine canyon where a depth of 1,436 m has been recorded off the town of Yelapa (Calderon Aguilera et al. 2009). This submarine canyon is several kilometres long and is located close to the coast. It has a steep slope that quickly reaches 90°, resulting in irregular, almost vertical rock walls with an accumulation of fine sediments. The average surface temperature in the Bay is 26.4°C, with a seasonal variation from 23.3°C (in March) to 30.0°C (in September). During winter, upwelling occurs, causing a decrease in surface temperature to 20°C and a shift of the thermocline from 40 to 20 m depth (Carriquiry et al. 2001).

### Sampling sites

**Figure 1.**
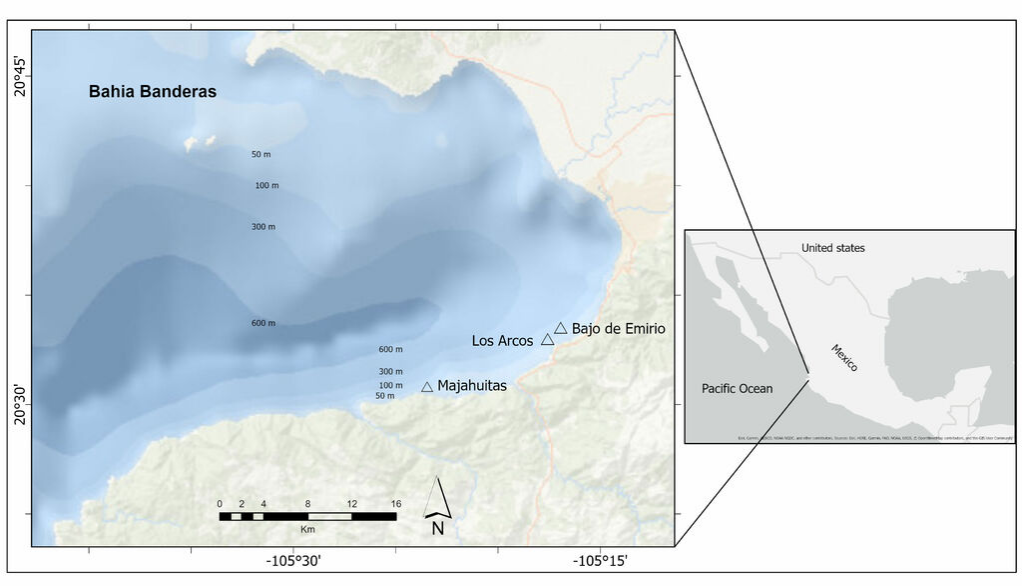
Study area showing sampling sites in Bahía de Banderas, Mexico.

**Figure 2.**
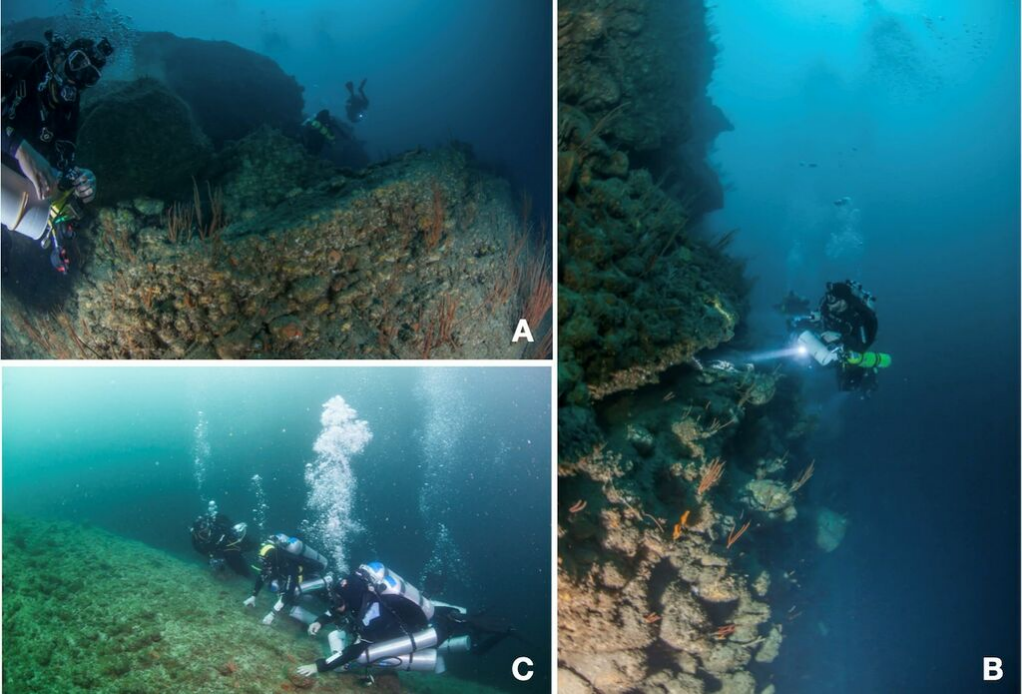
Images showing the geomorphology of the sampling sites. **A** Bajo de Emirio (BE); it is a wall formed by large stepped rocky platforms superimposed on each other; **B** Los Arcos (LA); a heterogeneous vertical wall formed by encrusted rocks of small size that form cavities and crevices; **C** Majahuitas (MH); there is a smoother slope with small and medium-sized boulders. In all three localities, there are important accumulations of sediments, also different species of sea fans and sponges are common. Photographs by Armando Perez Otegui.

Fish were sampled at three locations (Fig. 1): 1) El Bajo de Emirio (BE), which is part of the submarine canyon located south of the Bay, where the reef begins at 45 m depth with a rocky massif on a sandy slope. From this depth, the canyon has vertical walls with large boulders, some of which are superimposed, forming crevices and caverns (Fig. 2). The benthic habitat structure is dominated by coral fans. Fine to medium-grained sediment is present on the rocky ledges and can be easily removed. 2) Los Arcos Submarine Canyon (LA) is one of the best known and visited areas in the region. On its western edge, the canyon begins at the islet, which is a platform about 18 m deep, from which there are narrow passages with an inclination of about 45°; at 23 m, it becomes a vertical wall, with the presence of terraces at 50 and 70 m. However, most of these are walls formed by boulders embedded in the wall, forming bases for sea fans, sponges and other invertebrates (Fig. 2). Similar to El Bajo de Emirio, fine sediment is present here. 3) Majahuitas Beach (MH) is the south-westernmost point of the Bay and is a sandy beach that begins with a very steep slope immediately after the coastline with a gradient of about 60°. At the western end, a rocky reef begins formed by large boulders and pebbles. This morphology is maintained to a depth of about 80 m, where it becomes sandy. Unlike the other two sampling sites, this is not a vertical wall. Instead, there are some patches of substrate with medium to coarse-grained sand. Here, there is less coverage of sea fans and sponges, but a greater number of cracks and holes that serve as shelters for various organisms (Fig. 2).

## Data resources

### Fieldwork

Visual surveys were conducted along 10-minute banded transects at three depth levels (50, 60 and 70 m) at the three sites. Sampling was conducted between July and September in both 2018 and 2019. Five to six replicates (transects) were conducted at each site, with one or two transects per depth. However, because the total number of replicates was not equal, we decided to concentrate them into a single value for each site and depth for statistical analysis. During each visit, open circuit technical diving was conducted with gas mixes, using Trimix 18/40 as bottom mix and two Nitrox mixes of 50 and 80% O_2_ as decompression gas. The fish observed were identified *in situ* and the size per individual and abundance per species were estimated. In addition, a diver took underwater photographs and videos to later confirm identifications, for which several identification guides from Humann (1993), Allen and Robertson (1994), Gotshall (1998), Thomson et al. (2000) and Robertson and Allen (2015) were used. The order of the systematic list followed Nelson et al. (2016) and we confirm taxonomy with Eschmeyer's Catalog of Fishes (Fricke et al. 2023). Depth and temperature of each transect were recorded on a Shearwater Perdix AI dive computer. Bibliographic information was used to identify the morphofunctional characteristics of the species, their life history, bathymetric and geographic distribution and their local and national fisheries importance.

### Data analysis

#### Alfa Diversity

The data analysis considered a two-way crossed factorial design without replication, with depth and locality as factors according to the following model:

y = µ + Locality + Depth + (Locality*Depth) + e

To analyse alpha diversity, the four-step procedure proposed by Chao et al. (2020), based on Hill's numbers, was used with the iNext programme (http://chaostat.nthu.edu.tw/wordpress/software_download/) to calculate the following community metrics:

1) To assess the completeness profile of the sample, which includes both detected and undetected species, we estimated the proportion of the total number of individuals in a set that belongs to the species represented in the sample. This calculation was done using different values of q: q = 0 to determine the observed species, q = 1 to measure the diversity value, based on Shannon's index and q = 2 to represent the most abundant species, based on Simpson's diversity index.

2) Rarefaction and extrapolation analysis, based on the size and asymptotic diversity profile for q = 0, 1 and 2, were divided into two sub-analyses:

(a) The pattern of rarefaction and extrapolation size on the sample curve up to twice the sample size for indices of order q = 0, 1 and 2 to determine if the curve remains at a fixed value. The asymptote is used to infer the true diversity of the entire assemblage.

b) Based on the inference of true diversity, it is possible to determine the value of the undetected species by comparing the estimated asymptote to the empirical asymptote profile. The difference can then be evaluated and tested for significance.

3) Rarefaction based on non-asymptotic coverage and extrapolation analysis for order indices q = 0, 1 and 2. This was used to compare the diversity of fish assemblages between sites and depths. To do this, coverage data were standardised and compared with an integration of rarefaction and extrapolation, based on a maximum coverage value (Cmax).

4) Equitability profile. The magnitude of the normalised slope of the diversity profile was used to measure the equitability of species abundance by comparing the equitability to the Cmax value between sites and depths. The formula E = (D-1)/(S-1) in terms of q-order indices was used to calculate equitability, where diversity D and S were calculated with Cmax cover values. The Pielou index value J' was added to the Cmax cover values and standardised in the range (0, 1) to account for the effect of different specific richness.

#### Beta diversity

Comparison of species composition and abundance between sites and depths was performed with a two-way permutational analysis of variance (PERMANOVA) with crossed factors without replication, where each factor had a fixed effect (type I model). For this, the original data matrix had a fourth-root pretreatment and a Bray-Curtis similarity matrix was constructed. Statistical significance was tested with 10,000 residual permutations under a reduced model and with a type III sum of squares. Due to the small number of permutations, a posteriori tests were used for factors with significant differences between their levels, based on the Monte Carlo (MC) test.

The contribution of the average dissimilarity of fish species between locations and depths was estimated using the percentage similarity analysis (SIMPER). The SIMPER analysis was performed with the same pretreatment information and similarity measures were performed with the same pretreatment and similarity coefficient from PERMANOVA.

An non-metric multidimensional scaling (NMDS) analysis was performed to determine the spatial ranking between sites and sampling depths in terms of fish species detected. The same pre-treatment and similarity coefficient from PERMANOVA was also used.

A "shade plot" was used to show the change in fish species composition and abundance between depths and sampled locations. To associate samples (depth by location) in a Q-mode analysis, the data were pre-processed with a square root to estimate a Bray-Curtis similarity. To associate species in an R-mode analysis, Whitaker's association index was used with previously standardised relative abundance data. The dendrogram was constructed using the Unweighted Pair Group Method with Arithmetic Mean (UPGMA) method. Analyses (PERMANOVA, SIMPER, NMDS and shade plot) were performed in Primer v. 7 (Clarke and Gorley 2006) and PERMANOVA+ (Anderson et al. 2008).

## Results

### Alpha diversity

A temperature record was made during the two years of sampling in the different sites sampled, finding a constant temperature of 18 degrees Celsius from 40 m depth up to 70 m depth, where the sampling limit was reached. Visibility remained constant during the surveys at approximately 10 m. During these samplings, a total of 22 fish species belonging to 14 families were recorded in the three sampling sites (Table 1). The most representative family was Epinephelidae with five species, followed by Serranidae with three species and Labridae, Pomacentridae and Priacanthidae with two species each. Other families had only one species. El Bajo de Emirio (BE) presented 11 species, Los Arcos (LA) 12 species and Majahuitas (MH) was the site with the highest richness (21 species). A decline in total species richness was registered as depth increased, with 16 species observed at 50 m, nine species at 60 m and six species at 70 m. The most abundant species in BE was *Chromislimbaughi* with 50 specimens, whereas, in LA, *C.limbaughi* and *Haemulonmaculicauda* were the most abundant with 20 and 10 specimens, respectively. In MH, the same two species were present, although in this case *H.maculicauda* was the most abundant with 60 specimens, while *C.limbaughi* recorded 35 specimens. At 50 m depth, *C.limbaughi* was the most abundant with 75 specimens. *C.limbaughi* and *H.maculicauda* were the most abundant at 60 m. At 70 m, the most abundant species were *Chaetodonhumeralis* with 13 specimens and *Hyporthoduscifuentesi* with five specimens (Table 1).

### Assessment of sample completeness profile

The percent completeness estimated for each locality and depth was different. Diversity completeness q = 0 had low to moderate values between localities (67-81%) and between depths (59-87%). In contrast, values for q = 1 and q = 2 diversities were high (92-100%) for both localities and depths (Table 2). The lowest values for q = 0 correspond to the BE and the 50 m depth. In contrast, the analysis of abundant (q = 1) and very abundant (q = 2) species showed high completeness values with good representation of the samples obtained at the localities and depths studied. In other words, the undetected species with the highest percentage values correspond to BE with 33% and to the depth of 50 m with 41%. However, in the other localities and depths, relatively low percentages of the number of undetected species were maintained in the analysis by locality: LA 19%, MH 20%, while for the depth analysis, it was 13% for both 60 and 70 m (Table 2).

### Rarefaction analysis and extrapolation of q-order diversity

The asymptotic analysis of the localities showed that the diversity q = 1 and q = 2 were well represented by a value of less than one undetected species for each locality. However, for diversity q = 0 (species richness), there was not enough information to estimate the richness accurately, since the extrapolation percentage of undetected species was 29% for BE, 15% for LA and 18% for MH. On the other hand, the result of the analysis of rarefaction and extrapolation of diversity did not show a significant difference.

The asymptotic analysis shows that the diversity q = 1 and q = 2 for the three depths had a high representation of abundant and very abundant species, showing values less than one undetected species for each depth. On the other hand, the q = 0 was a good estimator of richness for the depths of 60 m (8% undetected species) and 70 m (2.18% undetected species), while for the depth of 50 m, it showed a high percentage of undetected species (39.8%) (Table 2).

### Rarefaction based on non-asymptotic coverage and extrapolation analysis

For species richness by site, although the data were insufficient to infer the true richness of the entire assemblage, inferences and significance tests can be made up to a standardised coverage value of C_max_ = 92.3%. At a standardised coverage of 92.3%, the estimated richness is 20 species for MH, 13 species for LA and 10 species for BE. Although the tests were successful, the richness shows low representation. The graphs show a significant difference between MH and the other two sites. The difference between MH and BE is 10 species, while the difference between MH and LA is seven species. For the diversity index q = 1, there was a difference of three species between MH and BE and only one species between MH and LA. The graphs show no significant difference between LA and MH localities, but there is a significant difference between LA and MH with respect to BE. For Simpson's diversity (q = 2), it showed that there is a greater dominance of abundant species in the localities of LA and MH with respect to what was found in the locality of BE, the graphs showing that there is a significant difference based on the confidence intervals (Table 2).

Regarding the species richness by depth, it can be concluded that, with a standardised coverage of 95.5%, the estimated richness is 16.0 for the depth of 50 m, 13.4 for 60 m and 6.7 for 70 m, where the graphs show a significant difference for 70 m with respect to 50 and 60 m. The difference between 70 and 50 m is 9.2 species, while the difference between 70 and 60 m is 6.6 species (Table 2). The difference between 70 and 50 m is 9.2 species, while the difference between 70 and 60 m is 6.6 species. For an assemblage fraction of 95.5%, the difference in diversity q = 1 between 70 and 50 m is one species and between 60 and 70 m < 1 species, with no apparent significant difference. In contrast, while for Simpson diversity (q = 2), these differ between 70 and 60 m of one species and between 70 and 60 m was almost two species, showing no significant difference between complete assemblages (Table 2).

### Evenness profiles

**Figure 3.**
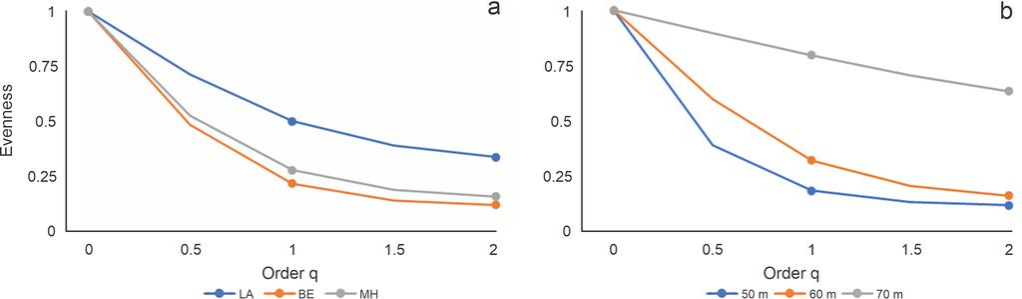
Evenness profiles calculated with the Hill numbers in the three orders of q = 0, 1 and 2, a) for each site (LA = Los Arcos, BE = Bajo de Emirio, and MH = Majahuitas) and b) depth (50, 60 and 70 m).

Analysis of the evenness profiles under the 92.3% cover value, the q = 0, q = 1, q = 2 and Pielou indices showed no significant difference at 95% confidence. On the other hand, the depth analysis using the 95.5% cover showed a pattern of increasing species evenness with increasing depth analysed, with the 70 m level being the one with the highest evenness, showing a significant difference with 95% confidence with respect to the other two depth levels (Table 2; Fig. 3).

### Beta diversity

PERMANOVA results showed no significant spatial variation in fish composition and abundance amongst sites, but significant variation amongst depth levels (Table 3). The total variation explained by the model was 56.9%. A posteriori tests showed that only depths 50 and 70 m had statistically different species dissimilarity (Table 4).

**Figure 4.**
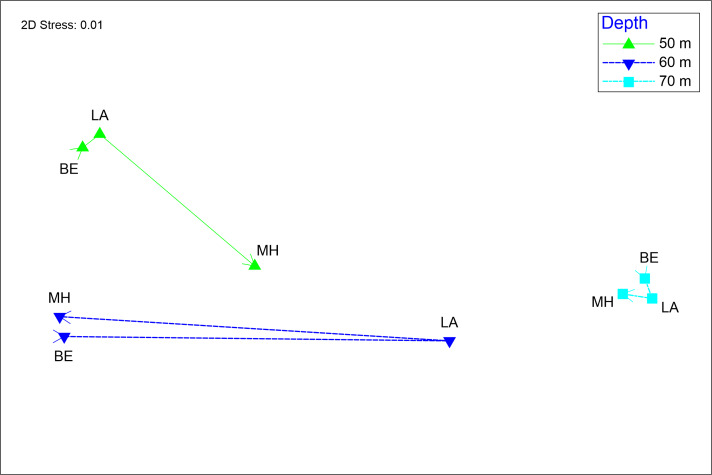
NMDS ordering showing the grouping of localities by depth (50, 60 and 70 m), organised by locality (BE = Bajo de Emirio; LA = Los Arcos and MH = Majahuitas).

SIMPER identified the species with the highest dissimilarity (95.7%) between the 50 m and 70 m depths as *Chromislimbaughi*, *Chaetodonhumeralis*, *Paralabraxmaculatofasciatus*, *Serranuspsittacinus*, *Hyporthoduscifuentesi* and *Haemulonmaculicauda* (Tables 5, 6, 7). The NMDS ordination showed a shift in fish species composition and abundance between depth levels, with the 70 m depth having the highest species similarity amongst the sites analysed, while the 50 and 60 m depths had greater variation in fish assemblage structure. At 50 m, the MH site had the greatest dissimilarity in species composition and abundance and, at 60 m, the LA site had the greatest dissimilarity (Fig. 4).

**Figure 5.**
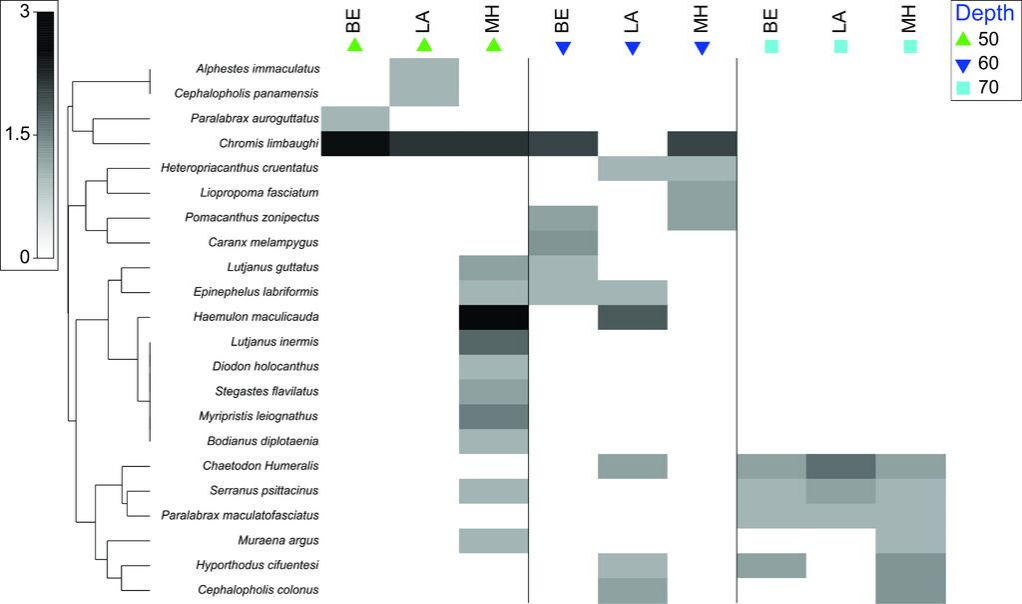
Shadow plots showing the fourth-root analysis and the Whittaker association index, using the UPGMA method (average group), the depth levels (50, 60 and 70 m), organised by locality (BE = Bajo de Emirio; LA = Los Arcos and MH = Majahuitas).

The shadow graph showed that, at 50 m, the only species recorded were *Alphestesimmaculatus*, *Bodianusdiplotaenia*, *Cephalopholispanamensis*, *Diodonholocanthus*, *Lutjanusinermis*, *Myripristiesleiognathus*, *Muraenaargus*, *Paralabraxaurogulatus* and *Stegastesflavilatus*. At a depth of 60 m, the species observed were *Heteropriacantuscruentatus*, *Liopropomafasciatum*, *Pomacanthuszonipectus* and *Caranxmelampygus*. Finally, the only species recorded exclusively at 70 m was *P.maculofasciatus* (Fig. 4). On the other hand, the species found at different depths were as follows: 1) between 50 and 60 m were *C.limbaughi*, *L.guttatus*, *E.labriformis* and *H.maculicauda*; 2) between 60 and 70 m were *C.humeralis*, *H.cifuentesi* and *C.colonus*; 3) between 50 and 70 m were *S.psittacinus* and *M.argus*. However, no species inhabiting the three depths studied were found (Fig. 5).

The analysis by site showed that, in BE, there were two exclusive species, *P.auroguttatus* at 50 m depth and *C.melampygus* at 60 m depth. In LA, two exclusive species were also observed at 50 m depth: *A.immaculatus* and *C.panamensis*. MH had the highest number of species not shared with other sites: *L.inermis*, *D.holocanthus*, *S.flavilatus*, *M.leiognathus* and *B.diplotaenia* at 50 m depth, as well as *L.fasciatum* at 60 m and *M.argus* at 50 m and 70 m (Fig. 5).

## Discussion

### Species richness

The mesophotic reefs of the southern Bahía de Banderas exhibited a richness of 22 fish species, representing 20% of the species reported in the entire Bay (Moncayo Estrada et al. 2006) and 40% of the fish species reported in the shallow reef of Los Arcos, on the eastern central coast of the same Bay (Stokes et al. 2019).

This difference in fish richness between shallow and mesophotic reefs has been reported in other regions (Puglise et al. 2009, Baker et al. 2016, Loya et al. 2019), including the Mexican Pacific, as recently demonstrated by Hollarsmith et al. (2020) with shallow and deep-water fish inventories in Bahía de La Paz, Baja California Sur, Mexico and Socorro Island in Revillagigedo, Colima, Mexico. However, the number of species reported in mesophotic reefs is highly variable. For instance, in the Caribbean, the range is low, between 19 and 32 species (Rosa et al. 2016) and Bahía de Banderas has 22 species; in contrast, Hawaii presents high richness with 148 species (Fukunaga et al. 2016), while oceanic islands in the Atlantic show as many as 158 species (Feitoza et al. 2005). Although the ichthyofauna of mesophotic reefs is not considered very rich, works with sufficient sampling effort provide comprehensive lists that include all species, visitors, residents, migrants and pelagics (Pyle et al. 2019, Hollarsmith et al. 2020).

The different works on mesophotic reefs in the world mention that the low richness is due to the presence of a thermocline, which serves as a barrier between the high richness shallow environment and the mesophotic with fewer species. Regarding temperature as a physical parameter, Bahía de Banderas registered a significant difference in temperature during sampling between the surface waters. Shallow waters ranged between 28 and 21°C, up to the thermocline detected at 40 m depth with a temperature of 18°C, which remained constant during the seasons in which the samples were collected. This provides evidence for the existence of a temperature parameter-based boundary for the mesophotic reef in Bahía de Banderas. Baker et al. (2016) and Bongaerts and Smith (2019) mention that finding transition zones is important for planning future research and creating management plans, as it allows the identification of species movements between the two zones, based on the characteristics of each environment (Bongaerts et al. 2010, Laverick et al. 2020).

### Endemism

A frequently-reported issue in mesophotic environments is the presence of endemic species and records of new species and geographic range extensions as a result of medium- and long-term projects conducted over an extensive bathymetric range (some greater than 100 m depth) (Kosaki et al. 1991, Pyle et al. 2008, Kane et al. 2014, Copus et al. 2015, Pyle et al. 2019). No endemic species, range extensions or new species were found in Bahia de Banderas, probably due to the sampling covering only a limited area.

### Decrease in species richness with increasing depth

Similar to other mesophotic reefs around the world (Pyle et al. 2019), the asymptotic profiles by depth show a significant difference between the three depth levels assessed. A decrease in observed and estimated species numbers was recorded with increasing depth, with the 50 m level having the highest richness with 16 species observed and the 70 m level having the lowest value with seven species. The record of declining species numbers is a constant in mesophotic studies (Brokovich et al. 2008, Serna Rodríguez et al. 2016, Fukunaga et al. 2017, Coleman et al. 2018, Williams et al. 2019, Pyle et al. 2019) that report a decline in species numbers with increasing depth, specifically Brokovich et al. (2008) recording this clear decline in richness in the Red Sea. This same pattern of decreasing fish species with increasing depth was reported by Fukunaga et al. (2016) in Hawaiian reefs, where they found a lower number of species in mesophotic reefs compared to shallow reefs, in addition to a higher number of endemics, while Pyle et al. (2019) mention this phenomenon in several studies around the world. For the studies conducted in Bahía de La Paz and Socorro Island in Revillagigedo, following this characteristic pattern of declining species numbers (Hollarsmith et al. 2020), in Bahía de Banderas, the same phenomenon of decrease of reduced species number is also observed. The factors that influence this change in the richness of mesophotic fish species are attributed to the availability of habitat, food and shelter (Pyle et al. 2019). In the mesophotic reefs of Bahía de Banderas, we observed a decrease in the presence of algae that support the herbivorous species of the shallow areas, just as we found species associated with colder waters. Additionally, the mesophotic environment of Bahía de Banderas presents a different morphology and substrate from the shallow one.

### Species composition in the mesophotic zone of Bahía de Banderas

The species recorded are part of a combination of species widely recognised as belonging to shallow reefs and species more associated with deep environments. In mesophotic studies, this information is crucial for recognising the boundary between the two environments. In a study conducted in Micronesia (Coleman et al. 2018), they reported a shift from the dominant shallow species that are herbivores to carnivorous and zooplanthophagous species that dominate the mesophotic zones. In this study, it was found that, in the three depth levels, the species were mostly carnivorous, such as *M.argus*, *A.immaculatus*, *E.labriformis*, *C.panamensis*, *S.psittacinus*, *P.maculofasciatus*, *P.aurogulatus*, *L.inermis*, *L.fasciatum*, *L.guttatus*, *H.maculicauda*, *H.cifuentesi*, *P.zonipectus*, *C.humeralis*, *B.diplotaenia* and *C.melampygus*. Two zooplanktivorous species were found: *C.colonus* and *C.limbaughi*; and two algivorous species: *D.holocanthus* and *S.flavilatus*, both corresponding to species recorded only at the 50 m level. At 70 m, carnivorous species dominated: *C.humeralis*, *M.argus* and *H.cifuentesi*. At 60 m, species of commercial interest belonging to the families Lutjanidae, Epinephelidae and Serranidae were found. In terms of conservation, only *P.zonipectus* was recorded, a species protected by the mandatory official Mexican standard NOM-059-SEMARNAT-2010, which makes it more valuable to consider the mesophotic zone for future conservation plans.

Changes in species composition with depth are unclear, but may be due to specialisation in feeding, behaviour or breeding season or the availability of refugia on the reef to protect them from predators (Coleman et al. 2018, Williams et al. 2019, Hollarsmith et al. 2020). The families Pomacentridae, Lutjanidae and Haemulidae were the most abundant in the shallowest 50 m and always formed clusters. On the other hand, *H.cifuentesi* and *L.fasciatum* were found exclusively at the 70 m level and can be considered as typical mesophotic species. Species contributing to the differences between the 50 and 70 m depth levels were identified as *C.limbaughi*, *C.humeralis*, *P.maculatofasciatus*, *S.psittacinus*, *H.cifuentesi* and *H.maculicauda*.

Other studies have described that the composition of carnivorous species dominates mesophotic environments and mainly because, in these studies, the mesophotic zone is associated with coral reefs that penetrate to this depth and favour food for carnivorous species (Fukunaga et al. 2016, Pyle et al. 2019). The same explanation is offered by Hollarsmith et al. (2020) for the area around Bahía de La Paz, Bja California Sur, Mexico and Socorro Island, Colima, Mexico in the Mexican Pacific. Bahía de Banderas does not have such a large coral composition for corals to be the main food source and fish assemblages are influenced by other food sources, which may explain why most of the species in the mesophotic zone of Bahía de Banderas are species commonly found on shallow reefs (Moncayo Estrada et al. 2006, Stokes et al. 2019).

### Beta diversity, species turnover by location

On the other hand, we did not find differences in fish assemblages amongst the Bahía de Banderas sites, which is not consistent with what has been reported in other publications, where differences in assemblages are recorded amongst nearby reefs of the Great Barrier Reef in Australia (Bridge et al. 2011). This may be due to the similarity of geomorphology across the Bahía de Banderas sites, In contrast, the reefs in Australia exhibit a great variety of morphology amongst mesophotic reefs due to the high richness of coral species that develop in those reefs, whereas in Bahía de Banderas, there is no such morphology of coral origin; instead, the three sampling areas share a common rocky bottom structure. The analysis of equitability did not show any significant difference between the sites. When looking at the equitability profiles in the results, it is observed how similar these profiles are between MH and BE.

Suitable habitats for organisms in the mesophotic zone depend on several factors, including light availability, substrate, temperature and other parameters (Puglise et al. 2009). In our case, we found a similarity in temperature and visibility (light penetration) between sampling sites. However, in the absence of an analysis aimed at assessing reef morphology, future studies are recommended to assess morphology and its effect on fish assemblages in Bahía de Banderas. Most studies of fish assemblages in mesophotic zones correspond to coral reefs where species of hermatypic corals extend to the depths of the mesophotic zone. These corals create complex structures with greater heterogeneity that provide shelter and food for the fish species that inhabit them (Garcia-Sais 2010, Bridge et al. 2011, Coleman et al. 2018). In the case of the mesophotic environment of Bahía de Banderas, there are no stony corals, so it is a more homogeneous environment on a steep slope composed of rocks and sand terraces, with the presence of octocorals and sponges. Therefore, the number of herbivorous or phytoplanctophagous species will be low. This is because plants and anglae are limited in deeper sea environments due to the lack of light.

No significant differences in the structure of the fish assemblages were presented between the sites, which allows us to assume that the structural characteristics of the environment described for each site are not determining factors to differentiate the sites, so it can be considered that the same mesophotic environment is present throughout the submarine canyon of southern Bahía de Banderas. We can say that it is less complex than similar ones in the Caribbean, the Hawaiian Islands, the Great Barrier Reef or the Red Sea (Feitoza et al. 2005, Bridge et al. 2011, Fukunaga et al. 2016, Pyle and Copus 2019).

### Work to be developed in future studies

Here, we report the first inventory of fishes associated with the mesophotic reef zone of southern Bahía de Banderas and, although obtained during a limited sampling period, it is a good representation of fish assemblages at three depth levels of this zone. This information is relevant for future decision-making in the development of sustainable management projects in the area due to the tourism and real estate development that is currently in full growth. The development of sustainable plans becomes fundamental because the mesophotic zone has been proposed as a refuge where several shallow reef zone species perform different activities, such as feeding, reproduction and temporary juvenile habitat (Puglise et al. 2009, Laverick et al. 2016, Shlesinger et al. 2018, Bongaerts and Smith 2019). Studies describing the structure of fish assemblages in the mesophotic zone are important for identifying the particular species that use this zone, particularly those of commercial interest. We found species, such as *H.cifuentesi*, *E.labriformis*, *L.guttatus* and *H.maculicauda* that are frequently caught in shallow waters; these species may use the mesophotic environment as a refuge. However, more research is needed to determine the functional use of these species in the mesophotic reef of the Bay.

## Conclusions

Previous studies in Bahía de Banderas, Mexico, had only focused on the ichthyofauna of the shallow waters. We found 22 species from 14 families in the upper mesophotic reefs (50-70 m) of the southern region of the Bay, including several commercially important species commonly caught in shallow waters. Although there were no major differences in fish assemblage composition between sites, species richness decreased with incressing depth. Each assemblage is a diverse mixture of species restricted to a particular location and depth, as well as species with a wide vertical range. This is an indication of the preference of fish species for certain depths and also of the possibility of mobility to and from shallow waters. From this perspective, it is important to generate information that contributes to the understanding of the structure and function of mesophotic reef fishes, providing information that will allow the development of management strategies in Bahía de Banderas.

## Figures and Tables

**Table 1.** Distribution and abundance (total number of individuals) of fishes recorded in the studied localities and depths of the mesophotic zone of Bahía de Banderas. Codes: LA = Los Arcos, BE = Bajo de Emirio, MH = Majahuitas.

		Locality										
		BE				LA			MH			
Depth (m)			50	60	70	50	60	70	50	60	70	Total
Muraenidae												
*Muraenaargus* (Steindachner 1870)									1		1	2
Holocentridae												
*Myripristisleiognathus* Valenciennes 1846									5			5
Serranidae												
*Paralabraxauroguttatus* Walford 1936			1									1
*Paralabraxmaculatofasciatus* (Steindachner 1868)					1			1			1	3
*Serranuspsittacinus* Valenciennes 1846					1			2	1		1	5
Epinephelidae												
*Alphestesimmaculatus* Breder 1936						1						1
*Cephalopholispanamensis* (Steindachner 1876)						1						1
*Epinepheluslabriformis* (Jenyns 1840)				1			1		1			3
*Hyporthoduscifuentesi* (Lavenberg & Grove 1993)					2		1				3	6
*Cephalopholiscolonus* (Valenciennes 1846)							2				3	5
Liopropomatidae												
*Liopropomafasciatum* Bussing 1980									2			2
Priacanthidae												
*Heteropriacanthuscruentatus* (Lacepède 1801)							1			1		2
Carangidae												
*Caranxmelampygus* Cuvier 1833				3								3
Lutjanidae												
*Lutjanusguttatus* (Steindachner 1869)				1					2			3
*Lutjanusinermis* (Peters 1869)									8			8
Haemulidae												
*Haemulonmaculicauda* (Gill 1862)							10		60			70
Chaetodontidae												
*Chaetodonhumeralis* Günther, 1860					2		2	7			2	13
Pomacanthidae												
*Pomacanthuszonipectus* (Gill 1862)				2						2		4
Pomacentridae												
*Chromislimbaughi* Greenfield & Woods 1980			35	15		20			20	15		105
*Stegastesflavilatus* (Gill 1862)									2			2
Labridae												
*Bodianusdiplotaenia* (Gill 1862)									1			1
Diodontidae												
*Diodonholocanthus* Linnaeus 1758									1			1
Total number of individuals			36	22	6	23	17	10	104	18	11	247
Total number of species			2	5	4	3	6	3	12	3	6	22
Total number of individuals by locality			64			50			133			
Total number of species by locality			11			12			21			

**Table 2.** Results of the analysis proposed by Chao et al. (2020). The four steps are shown. 1. Completeness profile for locality and depth. 2. Asymptotic analysis for locality and depth. 3. Coverage-based rarefactions and interpolations for locality and depth. 4 Species equity by site and depth. Locations: Los Arcos (LA), Bajo de Emirio (BE) and Majahuitas (MH). Depths: 50 m, 60 m and 70 m.

Sample completeness profiles by site and depth							
Locality	q = 0 (%)	q = 1 (%)	q = 2 (%)	Depth	q = 0 (%)	q = 1 (%)	q = 2 (%)
LA	81	92	99	50 m	59	95	99
BE	67	92	99	60 m	87	95	99
MH	80	97	100	70 m	87	97	99
Asymptotic analysis by site and depth							
BE	q = 0 (%)	q = 1 (%)	q = 2 (%)	50 m	q = 0 (%)	q = 1 (%)	q = 2 (%)
Asymptotic	14.1	3.02	1.63	Asymptotic	26.6	4.8	3
Empirical	10	2.68	1.62	Empirical	16	4.42	3
Undetected	4.1	0.34	0.01	Undetected	10.6	0.38	0
LA	q = 0 (%)	q = 1 (%)	q = 2 (%)	60 m	q = 0 (%)	q = 1 (%)	q = 2 (%)
Asymptotic	15.46	7.7	5.18	Asymptotic	15.9	5.1	2.5
Empirical	13	6.74	4.85	Empirical	14	4.66	2.48
Undetected	2.46	0.96	0.33	Undetected	1.9	0.44	0.02
MH	q = 0 (%)	q = 1 (%)	q = 2 (%)	70 m	q = 0 (%)	q = 1 (%)	q = 2 (%)
Asymptotic	24.47	6.74	3.81	Asymptotic	7.48	5.94	4.72
Empirical	20	6.27	3.75	Empirical	7	5.31	4.23
Undetected	4.47	0.47	0.06	Undetected	0.48	0.63	0.49
Non-asymptotic coverage-based rarefaction and extrapolation by site and depth							
Standardised coverage C_max_ = 92.3% Locality				Standardized coverage C_max_ = 95.5% Depth			
Locality	q = 0	q = 1	q = 2	Depth	q = 0	q = 1	q = 2
BE	10	2.68	1.62	50 m	16	4.42	3.00
LA	13	6.74	4.85	60 m	13.44	4.62	2.48
MH	20	5.85	3.69	70 m	6.77	5.17	4.13
Species equity by site and depth.							
Locality	Pielou J’	q = 1	q = 2	Depth	Pielou J’	q = 1	q = 2
BE	0.428	0.214	0.116	50 m	0.509	0.180	0.114
LA	0.725	0.499	0.335	60 m	0.705	0.319	0.158
MH	0.622	0.276	0.156	70 m	0.877	0.795	0.631

**Table 3.** Results of the PERMANOVA test where a significant difference is observed in the transects grouped by depth.

Sources	df	SS	EM	Pseudo-F	P-Value	permanentes	CV
Sites	2	2203.3	1101.6	0.49762	0.8484	6125	17.6
Depth	2	15502	7750.8	3.5011	**0.0355**	6196	39.3
Res	4	8855.3	2213.8				43.1
Total	8	26560					

**Table 4.** Result of the Pair-Wise Test, showing that there is a significant difference in the depths between the transects of 50 m and those of 70 m.

Groups	t	P-Value	perms	P(MC)
50 - 60 m	0.98352	0.4502	38	0.4541
50 - 70 m	3.0732	0.0996	34	**0.0382**
60 - 70 m	2.0775	0.1723	34	0.1047

**Table 5.** Dissimilarity between transects of 50 m and 60 m.

Groups 50 & 60 m	Average dissimilarity = 73.00					
	Group 50 m	Group 60 m				
Species	Av. Abund.	Av. Abund.	Av. Diss.	Diss/SD	Contrib. %	Cum. %
*Chromislimbaughi*	2.22	1.31	7.26	0.88	9.95	9.95
*Haemulonmaculicauda*	0.93	0.59	7.01	0.94	9.60	19.55
*Pomacanthuszonipectus*	0	0.79	6.72	1.16	9.21	28.76
*Heteropriacanthuscruentatus*	0	0.67	5.50	1.16	7.54	36.30
*Epinepheluslabriformis*	0.33	0.67	4.74	1.00	6.49	42.79
*Liopropomafasciatum*	0	0.40	3.54	0.62	4.85	47.64
*Caranxmelampygus*	0	0.44	3.52	0.62	4.83	52.47
*Lutjanusguttatus*	0.40	0.33	3.49	0.83	4.78	57.24
*Paralabraxauroguttatus*	0.33	0	3.44	0.66	4.71	61.95
*Cephalopholispanamensis*	0.33	0	3.21	0.66	4.40	66.35

**Table 6.** Dissimilarity between transects of 50 m and 70 m.

Groups 50 & 70 m	Average dissimilarity = 95.73					
	Group 50 m	Group 70 m				
Species	Av. Abund.	Av. Abund.	Av. Diss.	Diss/SD	Contrib. %	Cum. %
*Chromislimbaughi*	2.22	0	21.16	2.33	22.11	22.11
*Chaetodonhumeralis*	0	1.33	12.71	2.13	13.28	35.39
*Paralabraxmaculatofasciatus*	0	1	9.40	2.56	9.82	45.21
*Serranuspsittacinus*	0.33	1.06	8.43	1.30	8.80	54.01
Hyporthoduscifuentesi	0	0.84	7.33	1.15	7.66	61.67
*Haemulonmaculicauda*	0.93	0	4.55	0.66	4.76	66.43

**Table 7.** Dissimilarity between transects of 60 m and 70 m.

Groups 60 & 70 m	Average dissimilarity = 88.00					
	Group 60 m	Group 70 m				
Species	Av. Abund.	Av. Abund.	Av. Diss.	Diss/SD	Contrib. %	Cum. %
*Chromislimbaughi*	1.31	0	12.22	1.30	13.88	13.88
*Serranuspsittacinus*	0	1.06	9.60	4.92	10.91	24.80
*Paralabraxmaculatofasciatus*	0	1	8.98	7.27	10.20	35.00
*Chaetodonhumeralis*	0.40	1.33	8.83	1.39	10.03	45.03
*Pomacanthuszonipectus*	0.79	0	7.38	1.30	8.39	53.42
*Hyporthoduscifuentesi*	0.33	0.84	6.32	1.22	7.19	60.61
*Heteropriacanthuscruentatus*	0.67	0	6.04	1.30	6.86	67.47
